# The Global Burden of *Clostridioides difficile* Infections, 2016–2024: A Systematic Review and Meta-Analysis

**DOI:** 10.3390/idr17020031

**Published:** 2025-04-14

**Authors:** Rachel A. A. Akorful, Alex Odoom, Aaron Awere-Duodu, Eric S. Donkor

**Affiliations:** Department of Medical Microbiology, University of Ghana Medical School, Korle Bu, Accra P.O. Box KB 4236, Ghana; akorfulrachel@gmail.com (R.A.A.A.); alexodoom2018@gmail.com (A.O.); aawereduodu@gmail.com (A.A.-D.)

**Keywords:** *Clostridioides difficile*, incidence, risk factors, epidemiology, systematic review, healthcare-associated

## Abstract

**Background**: *Clostridioides difficile* infection (CDI) is a major cause of healthcare-associated infections globally. Understanding variations in CDI incidence and outcomes across settings, populations, and regions is important for guiding prevention strategies. **Aim**: The aim of this study was to determine the global epidemiology of CDI to better understand disease burden across settings and geographic regions. **Methods**: Relevant publications were identified through searches of major databases, including PubMed, Scopus, and Web of Science, published from 1 January 2016 through 24 July 2024. Random effects models were used to pool estimates, and 95% confidence intervals (CIs) were calculated. **Results**: A total of 59 studies, representing 24 countries across North America, Europe, the Asia–Pacific region, Latin America, and the Middle East, met the inclusion criteria. The incidence of CDI was highest in hospital-onset healthcare facility settings, with 5.31 cases/1000 admissions (95% CI 3.76–7.12) and 5.00 cases/10,000 patient-days (95% CI 3.96–6.15). Long-term care facilities reported 44.24 cases/10,000 patient-days (95% CI 39.57–49.17). Pediatric populations faced a greater risk, with 4.52 cases/1000 admissions (95% CI 0.55–12.17), than adults did at 2.13 (95% CI 1.69–2.61). Recurrence rates were highest for community-acquired CDI at 16.22%. The death rates for the CDI cases tracked for 30 days and of unspecified duration were 8.32% and 16.05%, respectively. **Conclusions**: This comprehensive review identified healthcare facilities, long-term care, pediatric populations, and North America as disproportionately burdened. This finding provides guidance on priority areas and populations for targeted prevention through antimicrobial stewardship, infection control, and surveillance.

## 1. Introduction

*Clostridioides difficile* infection (CDI) remains a significant concern for patients in healthcare settings and communities globally [1,2]. In developed countries, *Clostridioides difficile* (CD) is the most common cause of healthcare-associated (HA) diarrhea [2,3,4]. This bacterium, a gram-positive, anaerobic, spore-forming, toxin-producing bacillus found in the intestines of humans and animals, as well as in the environment, can lead to various health issues. The symptoms range from mild, such as diarrhea, to severe, such as toxic megacolon, and in some cases CDI may even lead to death [5]. CD is primarily transmitted through the fecal–oral route, with antibiotic therapy being the leading risk factor due to its disruption of the gut microbiota [6,7]. Other medications, such as proton pump inhibitors (PPIs) [6,7,8] and histamine-2 receptor antagonists (H2RAs), also increase CDI risk by reducing gastric acidity and impairing immune function [6]. Additional common risk factors include advanced age [9], co-morbidities [6], prolonged hospitalization, residence in long-term care facilities, and healthcare exposure [1,6,10,11].

There are significantly varying CDI prevalence rates reported globally. Research by Borren et al. showed CDI incidence ranging from 2.60 to 25.20 cases per 10,000 patient-days in Asia among hospitalized patients. Mortality rates in these regions were reported at 8.9% [12]. Similarly, South American studies reported CDI rates ranging from 8.3% to 41%, with incidence rates reaching 12.9 cases per 1000 admissions [13,14]. Additionally, in North America, particularly in the United States, the Centers for Disease Control and Prevention (CDC) in Atlanta, Georgia, reported 101.3 cases per 100,000 people. Of these, 51.2% were community-acquired (CA-CDI), and 50.1% were healthcare-associated (HA-CDI). Approximately 250,000 people are infected with CD annually in the United States, leading to approximately 14,000 deaths each year, according to the CDC [9]. These studies show that CDI is a major health issue in different parts of the world [15,16].

A systematic review by Balsells et al. [17] reported that the rate of CDI in healthcare facilities was 2.24 cases for every 1000 hospital admissions per year and 3.54 cases every 10,000 patient-days. In the community, the CDI incidence was recorded at 14.34 cases per 100,000 population, with CDI rates generally higher in North America. [17]. As years have passed since the original study by Balsells et al. [17], it has become important to track how CDI has been changing, especially with the potential evolution or emergence of strains such as riboypes 012, 023, 027, 014/020, 017, 056, 106, and 078/126, which complicate CDI treatment [16,18,19,20,21]. CDI is now not only a problem in hospitals but also in the wider community [2]. Recent estimates also show that 15–35% of cases recur after treatment, leading to serious health issues and increased healthcare costs [22]. This aspect of recurrence was not addressed in the study of Balsells et al., highlighting a gap in their systematic review. Our systematic review aims to analyze trends in the incidence of CDI across different age groups, recurrences, isolated ribotypes, and associated deaths from 2016–2024.

## 2. Materials and Methods

### 2.1. Preferred Reporting Items for Systematic Reviews (PRISMA) Guidelines

A thorough systematic review, conducted following the guidelines outlined by the Preferred Reporting Items for Systematic Reviews and Meta-Analyses (PRISMA), was carried out via web-based databases [23]. The PRISMA guidelines offer a detailed checklist and flow diagram to aid in identifying, screening, and evaluating records during the review process.

### 2.2. Search Strategy

In this systemic review, we conducted a comprehensive search of PubMed, Scopus, and Web of Science to gather relevant literature published from 1 January 2016 to 24 July 2024. The search was performed from 19 July 2024 to 24 July 2024. The search terms included MESH and keywords such as “*Clostridioides difficile*”, “*C. difficile*”, “*Clostridium difficile*”, “*Clostridium difficile* infections”, “*Clostridium difficile* colitis”, “*Clostridium difficile* infection”, “morbidity”, “disease”, “mortality”, “fatality”, and “death”. These specific databases were selected because they are commonly used and cover a wide range of scientific papers. Boolean operators such as “OR” and “AND” were added to enhance the search sensitivity.

### 2.3. Inclusion and Exclusion Criteria

A two-step screening process was used to find studies of interest. First, titles and abstracts were checked to remove irrelevant or duplicate studies. The full texts of the remaining studies were subsequently evaluated to determine their suitability on the basis of predefined inclusion and exclusion criteria.

Studies meeting the following criteria were considered for analysis: peer-reviewed papers published from 1 January 2016 to 31 July 2024 in English that focused on observational or preinterventional studies and national surveillance reports. Studies that involved any population such as pediatric, adult, and elderly populations across various healthcare settings (hospitals, intensive care units, internal medicine wards, long-term facilities, nursing homes, and communities) were considered. They were required to investigate outcomes related to CDI incidence, mortality rates, and recurrence while providing clear case definitions in line with current CDI surveillance methods (histological or laboratory diagnosis) or administratively coded CDI hospitalizations using the International Classification of Diseases (ICD) codes such as ICD-9-CM = 008.45 or ICD-10 = A40.7. Additionally, studies needed to report data on incidence rates and relevant details such as case numbers, study populations, or rates, to be eligible for inclusion in the analysis.

In the review process, studies published before 2016, those with repeated data or unclear case ascertainment methods or definitions, those limited to outbreak data, those focused on small areas, or those that used a narrowed study group selection process, were systematically excluded to ensure that the analysis focused on recent, clear, varied, and practical research. Reviews, case reports, brief communications and letters to editors, and studies not published in English were excluded.

### 2.4. Data Extraction

The study data were extracted by two reviewers (RAAA, AAD) via an Excel template. Any questions or disagreements were discussed between the reviewers to ensure accurate and reliable information. Microsoft Excel 2013 was used for effective data management and analysis.

The extracted data included details such as author(s), publication year, WHO region (North America (NA), Latin America (LA), Africa (AR), Western Pacific (WP), Europe (EU), Southeast Asia (SEA), and Eastern Mediterranean (EM), country, study duration, participant ages, CDI case ascertainment, laboratory confirmation methods, number of CDI cases, recurrences, mortality, and strain types.

For hospitalizations with ICD codes, the data included the number of cases where CDI was listed as the primary or secondary analysis. For data extraction, the team collected incidence rates on the basis of the recommended CDI surveillance case definitions.

Information on where the CDI was acquired, such as community-associated (CA) settings, healthcare-facility-associated (HCF) settings, hospital-onset healthcare-facility-associated (HO-HCF) settings, high-risk settings such as internal medicine (IM), general or medical intensive care units (ICUs), long-term care facilities (LTCFs) such as nursing homes (NHs), and unspecified locations, was also extracted.

To avoid bias towards large studies in the meta-analysis, data from multicenter publications were extracted per individual healthcare facility and counted as separate data points. Depending on the case definition used, the team extracted and standardized incidence metrics such as the number of CDI cases per 1000 admissions per year, incidence per year, incidence density per 10,000 patient-days, and cumulative incidence per 100,000 population per year. When the publications provided data for specific age groups, this information was also extracted.

In studies where data such as total admissions, total patient-days, or total populations were not directly provided, these values were calculated using available information such as the total number of CDI cases and the respective incidence rates.

### 2.5. Statistical Analysis

The meta-analysis was conducted using the meta package in R Studio version 4.3.3. Data were extracted from individual studies into a data frame including the sample size, event rate, and standard error for each study. Random-effects models were fit to pool incidence rates (cases per 1000 admissions or 10,000 patient-days) using the metaprop function. Heterogeneity was assessed using the I^2^ statistic. Forest plots were generated to visually display pooled rates and 95% confidence intervals. Subgroup analyses compared rates between settings (CA, HO-HCF, HCF) and age groups (pediatric, adult) using meta-regression. Publication bias was assessed using funnel plots and Egger’s test. Recurrence and case fatality rates were pooled and analyzed similarly using metaprop and associated meta functions for forest plots, heterogeneity, and publication bias.

### 2.6. Quality Assessment

Three investigators (RAAA, AO, AAD) used the Strengthening the Reporting of Observational Studies in Epidemiology (STROBE) checklist to evaluate the quality of the included observational studies. The STROBE checklist was designed to ensure that the key aspects of the study design and reporting were clearly presented. The checklist contains 17 items, with each item rated as “YES” (1 point) if the study provides sufficient information, “Maybe” (0.5 points) if the information is unclear or partially met, or “NO” (0 points) if the criteria are not met. The total score for each study was 17 points. Studies scoring between 13.6 and 17 points were considered to be of high quality, those scoring between 8.5 and 13.5 points were also considered to be of moderate quality, and studies scoring below 8.5 were considered to be of low quality. Any differences among the three reviewers were discussed to ensure consistent evaluation of the reporting quality of the included papers.

## 3. Results

### 3.1. Search Results and Overview of Included Studies

**Figure 1:**
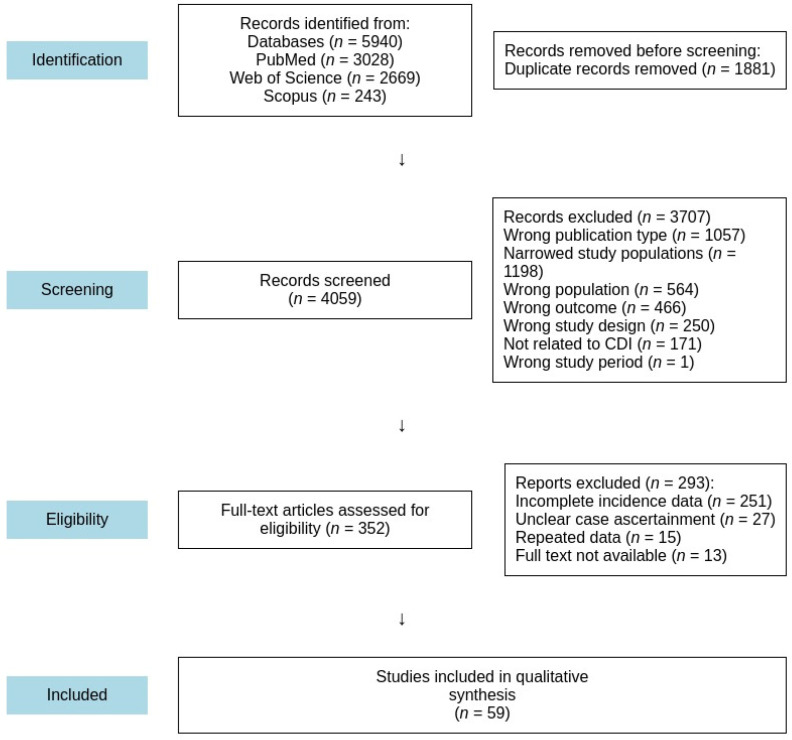
PRISMA flow diagram for the identification, screening, and evaluation of the articles included in the study.

The online database search resulted in 5940 publications. After removing duplicates, 4059 studies remained. The titles and abstracts of these studies were screened, and 3707 were excluded because they did not meet the inclusion criteria. The full texts of 352 studies were then assessed, of which 59 met the inclusion criteria (Figure 1). Of the 59 studies [24,25,26,27,28,29,30,31,32,33,34,35,36,37,38,39,40,41,42,43,44,45,46,47,48,49,50,51,52,53,54,55,56,57,58,59,60,61,62,63,64,65,66,67,68,69,70,71,72,73,74,75,76,77,78,79,80,81,82,83] included, 14 [27,34,36,37,38,41,42,45,51,62,63,67,69,71] were surveillance reports. Data extracted from each of the included studies are available (Table S1 in the Online Supplementary Document).

A total of 24 countries were included in the study, with most reports (42 out of 59) coming from NA [27,30,35,36,40,44,47,48,49,51,53,55,56,63,73,77,78,80,82] and EU [24,25,26,31,34,37,39,43,46,54,57,58,62,65,67,69,70,71,72,74,75,76,83], followed by WP (11/59) [28,29,32,33,42,52,59,60,61,66,68]. Reports from other regions such as LA [50], and EM [38,41,45,64,81] were less common. There was no country from Africa or Southeast Asia included.

With the CDI acquisition settings, 28 papers reported on HCF [24,29,30,31,32,34,37,42,47,48,50,51,53,54,57,58,60,61,65,67,68,69,72,75,76,80,81,83], 20 on HO-HCF [26,27,33,35,36,38,39,40,41,46,49,52,55,70,71,73,74,77,78,81], and 14 on CA [26,28,34,39,42,47,51,54,60,67,69,71,72,81]. Additionally, three [25,43,45] papers did not specify the CDI acquisition setting at all. In high-risk settings, nine [29,44,56,60,62,64,66,67,68], five [31,38,62,67,68], and four [31,62,63,82] papers reported on the ICU, IM, and LTCF settings, respectively. It is worth noting that the total count of articles reporting on various CDI acquisition settings exceeded the number of articles included in the review, as some articles addressed multiple settings concurrently.

Different age groups were considered. Most of the included studies focused on adults aged 18 years and older, including those aged 65 years and older. Some studies covered a wide age range, whereas others targeted specific groups. However, many studies did not provide clear age details.

The most reported ribotypes were RT027 and RT001 in several studies. Other ribotypes such as RT014, RT023, RT002, RT020, RT078, RT005, and RT018, NAP1 to NAP12, and RT028, RT015, RT016, RT017, RT046, RT085, RT106, RT126, RT176, RT193, RT026, and 356/607 were also found.

The studies looked at how people were diagnosed with and confirmed to have CDI. Some studies included laboratory results and symptoms together whereas some relied on just one. Some also relied on ICD codes (ICD-10 codes: A04.7 OR ICD: 008.45). The most common laboratory tests were glutamate dehydrogenase (GDH), enzyme immunoassays (EIAs) to find toxins A and B, and PCR, with some studies reporting on toxigenic culture. Most of the studies used a combination of these tests to ensure that the diagnosis was correct.

### 3.2. Meta-Analysis

#### 3.2.1. CDI Cases per 1000 Admissions

The number of CDI cases ranged from 0.10–5.31 per 1000 admissions. The highest numbers were found in the hospital-onset healthcare-facility-associated (HO-HCF) settings, with an average of 5.31 (95% CI = 3.76–7.12) cases per 1000 admissions. Healthcare-facility-associated (HCF) settings followed, with 4.26 (95% CI = 3.59–4.98) cases per 1000 admissions. On the other hand, community-associated settings (CA) had the lowest rate of 0.65 (95% CI = 0.07–1.74) per 1000 admissions. In different hospital settings, the general or medical intensive care units (ICUs) had 4.93 (95% CI = 1.40–10.56) cases per 1000 admissions, whereas those acquired from internal medicine (IM) wards had only 0.10 (95% CI = 0.04–0.19) cases per 1000 admissions. On average, across all of the settings, the overall estimate was 2.56 (95% CI = 2.18–2.96) per 1000 admissions (Table 1).

#### 3.2.2. CDI Cases per 10,000 Patient-Days

The number of CDI cases per 10,000 patient-days ranged from 0.67–44.24. The highest numbers were found in long-term care facility (LTCF) settings, with an average of 44.24 (95% CI = 39.57–49.17) per 10,000 patient-days. Internal medicine (IM) wards also presented high numbers, with 9.25 (CI = 5.28–14.27) cases per 10,000 patient-days, whereas healthcare-facility-associated (HCF) settings had 5.00 (95% CI = 3.96–6.15) cases per 10,000 patient days. For community-associated settings (CA), the rate was again lower at 0.67 (95% CI = 0.43–0.95) per 10,000 patient-days. When the data from all of the settings were combined, the overall estimate was 3.90 (3.34–4.51) per 10,000 patient-days (Table 1).

#### 3.2.3. CDI Cases per 100,000 Population

The number of CDI cases per 100,000 people (cumulative incidence) varied greatly across different settings, ranging from 20.62 to 96.60 per year. The highest rates were found in the hospital-onset healthcare-facility-associated (HO-HCF) settings, with 96.60 (95% CI = 20.12–230.18) cases per 100,000 people. Healthcare-facility-associated (HCF) settings also had high rates at 87.72 (95% CI = 2.93–289.70) per 100,000 people. Community-associated (CA) settings had the lowest rate at 20.62 (95% CI = 4.88–47.22). In nursing homes (NHs), the rate was 30.10 (95% CI = 20.25–41.89) per 100,000 people (Table 1).

#### 3.2.4. CDI Rates by Region

Among the different regions, North America (NA) had the highest number of CDI cases per 1000 admissions, with an average of 4.85 (95% CI = 4.07–5.70). Europe (EU) had 2.84 (95% CI = 1.76–4.18) cases per 1000 admissions, and the Western Pacific (WP) region had 2.34 (95% CI = 1.68–3.11) cases per 1000 admissions, with the Eastern Mediterranean (EM) region having the lowest number of 1.13 (95% CI = 0.04–3.64) cases per 1000 admissions (Table 2).

CDI cases per 10,000 patient-days were included. North America (NA) again had the highest rate at 6.23 (95% CI = 5.50–7.00), with Europe (EU) and the Western Pacific (WP) region showing 3.57 (95% CI = 2.73–4.52) and 3.59 (95% CI = 3.10–4.12) 10,000 patient-days, respectively (Table 2).

For the number of CDI cases per 100,000 people, North America (NA) also had the highest value at 66.02 (95% CI = 1.05–230.95), followed by Europe (EU) at 6.03 (95% CI = 23.86–50.68), with the Western Pacific (WP) region having a rate of 16.74 (95% CI = 4.30–37.29) per 100,000 people. The overall number of CDI cases across all regions was 43.49 (95% CI = 19.51–76.96) per 100,000 people (Table 2).

#### 3.2.5. CDI Rates by Age Group

For the number of CDI cases per 1000 admissions, the overall average was 2.56 (95% CI = 2.18–2.96). The highest rates were found in children, with a CDI rate of 4.52 (95% CI = 0.55–12.17) per 1000 admissions, whereas adults had a lower rate of 2.13 (95% CI = 1.69–2.61) per 1000 admissions.

For CDI cases per 10,000 patient-days, children again had the highest rates at 7.01 (95% CI = 5.81–8.31) whereas adults had 3.74 (95% CI = 2.83–4.78).

The number of CDI cases per 100,000 people was highest among adults, with an average of 34.20 (95% CI = 16.48–58.31). The overall number of CDI cases across all age groups was 43.49 (95% CI = 19.51–76.96) per 100,000 people (Table 3).

#### 3.2.6. CDI Recurrence Rates

**Figure 2:**
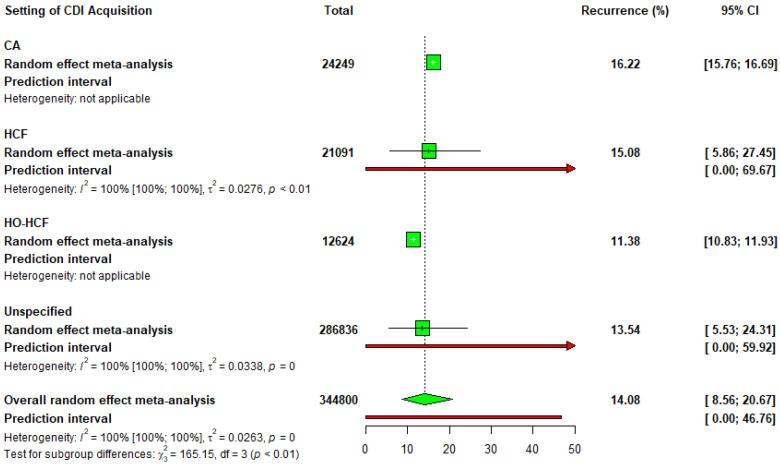
A forest plot of *C. difficile* infection recurrence rates across different settings of CDI acquisition. Green squares (□) represent the point estimates of recurrence rates for each subgroup in the meta-analysis. The green diamond (◊) indicates the overall pooled estimate from the random-effects meta-analysis. Red horizontal lines (-) represent the 95% confidence intervals (CI) for the recurrence rates, while the red arrows (→) highlight prediction intervals, showcasing the range within which the true effect size of future studies might fall. The horizontal axis displays the percentage of recurrence rates. Text annotations include heterogeneity statistics (I^2^, τ^2^, and *p*-values) and subgroup analysis results.

Recurrence rates of CDI (rCDI) were taken across the settings. Community-associated (CA) settings had the highest recurrence rate at 16.22% across 24,249 cases with low variability, whereas healthcare-facility-associated (HCF) settings had a recurrence rate of 15.08% from 21,091 cases with considerable variation between studies. The hospital-onset healthcare-facility-associated (HO-HCF) setting had the lowest recurrence rate of 11.38% from 12,624 cases, whereas the recurrence rate in unspecified settings was 13.54% from 286,836 cases, with considerable variation and a wide range of possible rates. In total, CDI recurred in 14.08% of 344,800 people. There was significant variation in recurrence rates between different studies with the highest rates occurring in community settings and the lowest rates occurring in hospital settings (Figure 2).

#### 3.2.7. CDI Case Fatality Rate (CFR)

**Figure 3:**
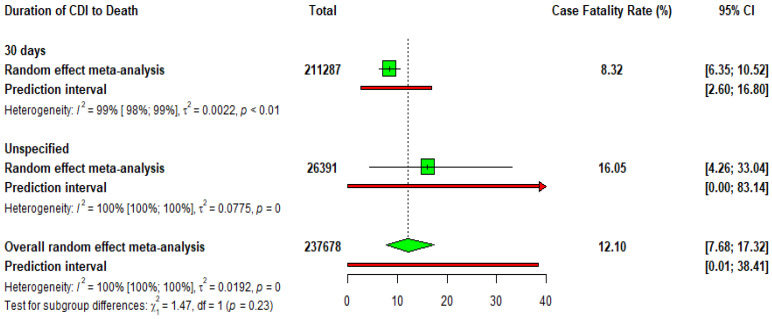
A forest plot of the duration of *Clostridioides difficile* infection to death. Green squares (□) represent the point estimates of case fatality rates for each subgroup in the meta-analysis. The green diamond (◊) indicates the overall pooled estimate from the random-effects meta-analysis. Red horizontal lines (-) represent the 95% confidence intervals (CI) for the case fatality rates, while the red arrows (→) highlight prediction intervals, showcasing the range within which the true effect size of future studies might fall. The horizontal axis displays the percentage of case fatality rates. Text annotations include heterogeneity statistics (I^2^, τ^2^, and *p*-values) as well as subgroup analysis results.

Death rates in the form of CFRs were determined for CDI based on the duration of the infection. For cases tracked for 30 days, the death rate was 8.32% out of 211,287 cases, with high variability between studies. For cases where the duration was unspecified, 16.05% of 26,391 cases resulted in death, and these results showed extreme variability. Overall, the combined death rate for CDI was 12.10% of 237.678 cases. There was a significant variation in death rates across different studies (Figure 3).

#### 3.2.8. Quality of the Included Studies

Most of the studies included (*n* = 41, 69.49%) were of high quality after the quality of the included papers was assessed via the STROBE checklist, whereas some (*n* = 18, 30.51%) of the other studies were of moderate quality. Importantly, no study was of low quality, indicating that overall, the research standards were good. The study size and bias were mostly not discussed across almost all of the studies (Table S2 in the Online Supplementary Document).

## 4. Discussion

Our findings demonstrate that community-acquired CDI had the lowest incidence across all measures at 0.65 per 1000 admissions, 0.67 per 10,000 patient-days, and 20.62 per 100,000 population. In contrast, healthcare-facility-onset CDI demonstrated a substantially greater incidence, exceeding 4 per 1000 admissions and almost 5 per 10,000 patient-days. Consistent with the findings of past systematic reviews, the incidence of community-acquired CDI was significantly lower than that of healthcare-associated disease [17,84]. Despite the relatively low incidence, Balsells et al. reported a community-acquired CDI incidence of 14.34 cases per 100,000 population for the years 2005 to 2015, which is lower than our present estimate of 20.62 cases for the years 2016–2024. This signifies that community risk is increasing, with a 43.8% increase from 2016–2024. Similarly, for healthcare transmission, they reported an incidence of 2.24 cases per 1000 admissions, which is less than our estimate of over 4, with a 90.2% increase from 2016–2024. Differences in incidence between settings appear to be amplifying over time, indicative of evolving epidemiology.

This heightened incidence observed in healthcare settings can be attributed to the fact that these environments inherently involve substantial antibiotic utilization, which is understood to disrupt the normal gut microbiota and increase the risk of *C. difficile* spore germination and overgrowth [7]. However, nosocomial transmission via contact with contaminated environmental surfaces or medical equipment or exposure to colonized or actively infected patients has been indicated as driving the in-facility amplification of cases [85,86]. The elevated incidence observed across healthcare settings likely reflects greater selective pressure imposed by hypervirulent *C. difficile* strains, such as the pandemic BI/NAP1/027 genotype, which dominate nosocomial infections but have also spread more extensively in some communities [87]. Frequent hospital readmissions may also increase the risk of recurrent pathogen exposure [39]. On the other hand, community-acquired *C. difficile* seems to depend more heavily on person-to-person or environmental reservoirs, challenging health systems’ efforts to curb incidence through contact precautions alone [88]. The epidemiologic drivers likely differ between healthcare and community settings, necessitating tailored prevention and control strategies, as well as investigation of the factors underlying the observed variations.

Among healthcare settings, hospital-onset healthcare facility (HO-HCF) CDI had the highest incidence per 1000 admissions at 5.31. The incidence of intensive care unit (ICU)-associated CDI was also high at 4.93 per 1000 admissions, which is comparable to that of HO-HCF. This high incidence in hospitals and ICUs can be attributed to these settings concentrating the most immunocompromised patients who are reliant on extensive medical intervention. These patients are at optimal risk of opportunistic pathogens, including *C. difficile*, due to the healthcare-associated influence on sanitation and the constant introduction of new cases. Before the COVID-19 pandemic, CDI was already a concern due to prolonged hospitalizations, mechanical ventilation, and widespread antibiotic use. However, during the pandemic, the incidence of CDI increased among COVID-19 ICU patients, with some hospitals reporting a threefold increase. This rise can be attributed to increased antibiotic use, longer stays, and hypervirulent C. difficile strains. These trends highlight the evolving risk profile for CDI in ICU settings and the need for robust infection control and antibiotic stewardship strategies [8]. Conversely, internal medicine wards exhibited a remarkably low CDI incidence of just 0.10 per 1000 admissions, suggesting that antimicrobial stewardship and infection prevention may be more optimized in these lower acuity areas. Notably, long-term care facilities and nursing homes emerged as high-risk settings, with LTCFs reporting an alarming rate of 44.24 CDI cases per 10,000 patient-days. The prolonged nature of chronic care for elderly, debilitated populations amid endemic healthcare-associated infections fosters constant selective pressure. Additionally, underlying comorbid conditions, such as inflammatory bowel disease or diabetes, and compromised immune defenses tend to be more common among older patient populations within hospital and long-term care facility networks, which are correlated with increased susceptibility to *C. difficile* infections [89].

Our findings on CDI recurrence rates provide useful insights into differences in the acquisition setting. Notably, community-associated CDI demonstrated the highest recurrence rate of 16.22%; however, our results contrast with those of the review by Finn et al. (2021) [84], where the rate of recurrence was lower for community-associated diseases. Timely diagnosis and extended antibiotic courses have been shown to lower recurrence [90,91] but may be more difficult to achieve outside structured inpatient care, leading to high recurrence of community-associated CDI. The lower 11.38% recurrence for hospital-onset healthcare facility cases aligns with expectations given intensive surveillance and infection prevention practices in these controlled settings. The overall 12.1% combined CFR confirms that CDI poses a serious threat, with over 1 in 10 cases ending in death. This underscores the need for timely, effective clinical management.

This meta-analysis also provided valuable insights into geographical differences in reported CDI incidence worldwide. Notably, North America presented the highest incidence per 1000 admissions (4.85) and per 10,000 patient-days (6.23), which was significantly greater than that reported in other regions. This is unsurprising given that the hypervirulent BI/NAP1/027 *C. difficile* lineage originated and propelled North America’s “first wave” epidemic in the 2000s [92,93]. Furthermore, increasing antimicrobial resistance in North American healthcare systems has resulted in increased selection of multidrug-resistant *C. difficile* strains [94]. Our results corroborate previous global findings indicating heavier CDI burdens in North American healthcare [17]. Europe also reported relatively elevated incidence rates of 2.84 and 3.57, respectively, although individual nations demonstrate divergent epidemiological characteristics. These higher incidences align with Europe, which represents developed nations with stringent reporting protocols [84]. In contrast, the Eastern Mediterranean region presented the lowest recorded incidences, at 1.13 and 1.42, respectively. However, data scarcity from this region likely underestimates the true disease burden.

Our observations indicate that developed regions experience greater burdens associated with *Clostridioides difficile* infection (CDI), while the incidence is increasing most rapidly in expanding global healthcare systems. Several interacting factors likely contribute to the elevated burden. First, more stringent mandatory reporting requirements and the availability of extensive molecular testing technologies in North America and Europe potentially facilitate superior disease monitoring and identification of symptomatic and asymptomatic cases. Second, reporting standards for CDI are uniform in these nations via regulations such as the CDC’s National Healthcare Safety Network and ECDC guidelines. Furthermore, developed areas in North America and Europe commonly provide universal healthcare access and elevated inpatient bed availability. This results in additional exposure opportunities and lengthened hospitalizations that further sustain susceptibility to nosocomial transmission. Finally, overprescription of broad-spectrum antibiotics has historically been more prevalent in wealthier markets as well, disrupting the gut microbiota and selecting for multidrug-resistant strains [7]. On the other hand, underreporting remains challenging in resource-limited settings, potentially explaining the lower reported incidence in Latin America, the Eastern Mediterranean region, and the Western Pacific region. Comparatively, less stringent prescribing in developing regions offers reduced selection pressures. Nevertheless, strengthened infrastructure in regions will likely drive gradual increases in incidence, especially with rapid urbanization and lifestyle modifications, potentially increasing transmission dynamics.

In addition to the variation in CDI rates across regions and settings, ribotype distribution plays a crucial role in disease outcomes. The most frequently reported ribotypes in the studies reviewed were RT027 and RT001, along with other ribotypes such as RT014, RT023, RT002, RT078, and several others, including NAP1 to NAP12. Ribotype RT027, in particular, is associated with increased severity and recurrence of CDI, especially in healthcare settings [16]. The distribution of these ribotypes highlights the need for targeted surveillance and infection control strategies, as certain strains may contribute to more severe outcomes and greater transmission risks [95]. These findings underscore the importance of ribotype surveillance in guiding prevention and treatment measures.

Our meta-analysis reveals important new insights into the disparity in *Clostridioides difficile* infection (CDI) incidence between pediatric and adult patient populations. Most notably, the incidence of CDI per 1000 admissions was substantially greater in children (4.52) than in adults (2.13), corroborating prior reviews [96,97] and underscoring the ongoing threat posed to hospitalized young patients. However, the actual rates of clinically significant CDI in these younger age groups are comparatively low. This suggests that testing and diagnosis of CDI in pediatric settings may often identify asymptomatic colonization rather than true disease, potentially overstating the true incidence. A likely explanatory factor for elevated pediatric inpatient CDI is differences in the gut microbiota composition and colonization resistance between age groups. The developing infant microbiome evolves rapidly in early life through environmental influences such as birth mode, feeding, antibiotic exposure, and hygiene—leaving it more vulnerable to pathogen overgrowth until stabilizing colonization [98]. Immune immaturity may also influence susceptibility, as innate and adaptive defenses continue strengthening throughout childhood. *C. difficile* toxins and spores may encounter less effective gastrointestinal clearance. We also found that the incidence measured by patient-days likewise emerged to be greater in children (7.01) than in adults (3.74), emphasizing longer admissions as a key risk period. Although our results compellingly demonstrate an appreciably elevated CDI risk for children during hospitalization compared with adults, heterogeneity was considerably greater between pediatric studies, introduced by variable case definitions, diagnostic practices, and outbreak criteria, obscuring true incidence. Younger patients additionally experience more diverse enteric pathogen-linked diarrhea, complicating etiological attribution. Therefore, harmonizing surveillance standards across pediatric centers remains a top priority.

Some limitations must be acknowledged. First, some otherwise relevant studies had to be excluded because they reported population-level CDI incidence in nonstandard formats, making it difficult to accurately calculate the parameter of interest needed for inclusion in the review. Additionally, most data came from North America, Europe, and Asia, with scarce evidence from other regions, such as Africa, complicating the analysis of global trends. Significant between-study heterogeneity precluded direct comparisons. Furthermore, variation in clinical definitions, including inconsistent confirmation of CDI versus colonization across settings, diagnostic methodologies, and reported outcomes, could alter pooled estimates.

## 5. Conclusions

This systematic review and meta-analysis offers crucial insights into the global epidemiology of *Clostridioides difficile* infection (CDI) by synthesizing data from over 50 studies across 24 countries. The highest CDI incidence occurs in healthcare facilities and long-term care facilities, emphasizing the role of healthcare exposure in transmission. Pediatric populations face greater risk than adults, indicating the need for tailored prevention strategies. Regional variations were noted, with North America reporting the highest incidence rates. Differences in recurrence and death rates were observed on the basis of the acquisition source and follow-up duration. Targeted strategies such as antimicrobial stewardship and surveillance are crucial for mitigating rising trends. While this study provides valuable insights, limitations such as heterogeneity and data gaps suggest the need for standardized monitoring to enhance epidemiologic understanding and inform policymaking.

## Figures and Tables

**Table 1:** Meta-analysis results for the incidence rates of CDI by setting of acquisition.

Setting of CDI Acquisition	Incidence of CDI per 1000 Admissions [95% CI]	Incidence of CDI per 10,000 Patient-Days [95% CI]	Incidence of CDI per 100,000 Population [95% CI]
Community-Acquired (CA)	0.65 [0.07; 1.74]	0.67 [0.43; 0.95]	20.62 [4.88; 47.22]
Healthcare Facility (HCF)	4.26 [3.59; 4.98]	5.00 [3.96; 6.15]	87.72 [2.93; 289.70]
Hospital Onset, Healthcare Facility (HO-HCF)	5.31 [3.76; 7.12]	3.79 [2.52; 5.31]	96.60 [20.12; 230.18]
Intensive Care Unit (ICU)	4.93 [1.40; 10.56]	3.24 [0.00; 21.80]	-
Internal Medicine (IM)	0.10 [0.04; 0.19]	9.25 [5.28; 14.27]	-
Long-Term Care Facility (LTCF)	-	44.24 [39.57; 49.17]	-
Nursing Home (NH)	-	-	30.10 [20.25; 41.89]
Unspecified	0.59 [0.36; 0.88]	4.29 [3.54; 5.09]	45.20 [17.64; 85.49]
Pooled Incidence Rate [95% CI]	2.56 [2.18; 2.96]	3.90 [3.34; 4.51]	43.49 [19.51; 76.96]

**Table 2:** Meta-analysis results for incidence rates of CDI by WHO region.

Continent	Incidence of CDI per 1000 Admissions [95% CI]	Incidence of CDI per 10,000 Patient-Days [95% CI]	Incidence of CDI per 100,000 Population [95% CI]
Eastern Mediterranean	1.13 [0.04; 3.64]	1.42 [0.55; 2.70]	-
Europe	2.84 [1.76; 4.18]	3.57 [2.73; 4.52]	36.03 [23.86; 50.68]
Latin America	1.69 [1.54; 1.84]	3.09 [2.82; 3.37]	-
North America	4.85 [4.07; 5.70]	6.23 [5.50; 7.00]	66.02 [1.05; 230.95]
Western Pacific	2.34 [1.68; 3.11]	3.59 [3.10; 4.12]	16.74 [4.30; 37.29]
Pooled Incidence Rate [95% CI]	2.56 [2.18; 2.96]	3.90 [3.34; 4.51]	43.49 [19.51; 76.96]

**Table 3:** Summary of global meta-analysis results for incidence of CDI in several categories stratified by age group.

Age Group	Incidence Rate [95%CI]	95% Prediction Interval	Number of Studies	Number of CDI Cases	H [95%CI]	I^2^ [95%CI]	P Heterogeneity
Incidence of CDI per 1000 Admissions							
Overall	2.56 [2.18; 2.96]	[0.24; 7.21]	30	206,749	29.49	99.9	0
Adults	2.13 [1.69–2.61]	[0.02; 7.28]	16	94,538	25.03	99.8	0
Children	4.52 [0.55–12.17]	[0.00; 383.83]	3	398	9.11 [7.00; 11.84]	98.8 [98.0; 99.3]	<0.0001
Incidence of CDI per 10,000 Patient-Days							
Overall	3.90 [3.34–4.51]	[0.39; 10.88]	26	160,055	27.56	99.9	0
Adults	3.74 [2.83–4.78]	[0.10; 12.04]	6	1735	5.48 [5.02; 5.98]	96.7 [96.0; 97.2]	<0.0001
Children	7.01 [5.81–8.31]	NA	2	936	1.11	19.3	0.2657
Incidence of CDI per 100,000 Population							
Overall	43.49 [19.51; 76.96]	[0.00; 349.60]	7	430,044	162.44	100.0	0
Adults	34.20 [16.48; 58.31]	[0.00; 168.87]	4	159,001	114.81	100.0	0

## Data Availability

All of the supporting data are presented in the manuscript and Supplementary Files.

## Supplementary Materials

The following supporting information can be downloaded at: https://www.mdpi.com/article/10.3390/idr17020031/s1, Table S1: Summary of Studies on *Clostridium difficile* Infection (CDI) Across Regions; Table S2: Assessment of Study Quality for *Clostridium difficile* Infection.
