# Supplementary material for: The Global Burden of Clostridioides difficile Infections, 2016–2024: A Systematic Review and Meta-Analysis
Source: Infect Dis Rep. 2025 Apr 14;17(2):31. doi: 10.3390/idr17020031 (PMC12026862; doi:10.3390/idr17020031)
Supplement: Supplementary file 1 [file idr-17-00031-s001.zip › idr-3282863-supplementary.pdf]

Supplementary Material: Akorful et al. Global Burden of *Clostridium Difficile* Infections 2016-2024: A Systematic Review and Meta-Analysis

Supplementary Table 1. Summary of Studies on *Clostridium difficile* Infection (CDI) Across Regions

Region	Reference	Country	Study yrs (if <1 year: mo/wk)	Age (years)	CDI ascertainment	Laboratory confirmation	CDI TYPE	Cases	Recurrence	100,000 populations	1,000 admissions	10,000 patient-days	Mortality	Strain
EU	Govoni (2020)	Bulgaria	2018-2019	>75	ICD-9-CM code 00845 + Lab	GDH	HCF	33	4		1192		7	
EU	Lauda-Maillen (2019)	France	2014-2015	<75 and ≥75	Lab+symp	GDH, PCR	HCF	101	23			1,123,456		
NA	Olsen (2019)	US	2010-2012	>=65	ICD-9-CM		LTCF	174,903		1,493,441				19,064 (30d)
NA	Miller (2016)	US	2005-2011	>18	ICD-9-CM code 008.45		HO-HCF	35,170		22,312,704				
NA	Longtin (2016)	Canada	2004-2013	No details	Lab+symp	GDH, EIA Tox A&B, Cyt, PCR	HO-HCF	722				600,358		NAP1
NA	Miller (2016)	US	2009-2011	No details	ICD code 008.45		HCF	215,571		23,634,793				
EM	Alkhawaja 1 (2024)	Bahrain	2021-2023	No details	Lab	GDH, PCR	HO-HCF	36			125,000	711,466		
	Alkhawaja 2 (2024)	Bahrain	2021-2023	No details	Lab	GDH, PCR	CA	18			125,000			
	Alkhawaja 3 (2024)	Bahrain	2021-2023	No details	Lab	GDH, PCR	HCF	39			125,000	709,090		
EU	Karaaslan (2016)	Turkey	2012-2014	<18	Lab+symp	EIA Tox A&B, Cyt	HCF	100			12196			
EU	Barbosa-Martins (2022)	Portugal	2013-2018	>=18	Lab+symp	EIA Tox A&B, PCR	unspecified	50			103,545			
EU	Kotila 1(2016)	Finland	2008-2013	All	Lab	NAAT, Cul	HO-HCF	16,319		32,349,544				RT027,RT001,RT014,RT023,RT002,RT020,RT078,RT005,RT018,RT011
	Kotila 2(2016)	Finland	2008-2013	All	Lab	NAAT, Cul	HCF	22,348		32,349,544		69,837,500	441(30 d)	RT027,RT001,RT014,RT023,RT002,RT020,RT078,RT005,RT018,RT011

	Kotila 3(2016)	Finland	2008-2013	All	Lab	NAAT, Cul	CA	10,643		32,349,544			106(30d))	RT027,RT001,RT014,RT023,RT002,RT020,RT078,RT005,RT018,RT011
NA	Katz 1 (2018)	Canada	2009	All	Lab	PCR	HO-HCF	1,627				2,757,627	13	NAP1-12
	Katz 2 (2018)	Canada	2010	All	Lab	PCR	HO-HCF	2,307				3,781,967	26	NAP1-12
	Katz 3 (2018)	Canada	2011	All	Lab	PCR	HO-HCF	2,795				4,171,642	36	NAP1-12
	Katz 4 (2018)	Canada	2012	All	Lab	PCR	HO-HCF	2,645				4,336,066	24	NAP1-12
	Katz 5 (2018)	Canada	2013	All	Lab	PCR	HO-HCF	2,746				5,280,769	21	NAP1-12
	Katz 6 (2018)	Canada	2014	All	Lab	PCR	HO-HCF	2,593				5,893,182	22	NAP1-12
	Katz 6 (2018)	Canada	2015	All	Lab	PCR	HO-HCF	2,489				5,788,372	16	NAP1-12
WP	Chen 1 (2017)	Australia	2006-2012	45-64	ICD-10-AM code A04·7	No details	CA	54		163,729				
	Chen 2 (2017)	Australia	2006-2012	>=65	ICD-10-AM code A04·7	No details	CA	133		103,193				
	Chen 3 (2017)	Australia	2006-2012	>=45	ICD-10-AM code A04·7	No details	CA	187		266 922				
WP	Xu 1 (2017)	China	2009-2016	>=18	Lab+symp	Cul, MALDI-TOF,PCR	HCF	307	13		910000		32 (30d)	RT 017,RT027,RT023,RT078,RT012,RT046,RT085,RT014,RT014
	Xu 2 (2017)	China	2009-2016	>=18	Lab+symp	Cul, MALDI-TOF,PCR	HCF, IM	43			910000			RT 017,RT027,RT023,RT078,RT012,RT046,RT085,RT014,RT015
	Xu 3 (2017)	China	2009-2016	>=18	Lab+symp	Cul, MALDI-TOF,PCR	HCF, ICU	58			910000			RT 017,RT027,RT023,RT078,RT012,RT046,RT085,RT014,RT016
WP	Kimura 1(2020)	Japan	2008-2017	>=18	Lab, ICD-10 codes (A04.7)	EIA:GDH, Tox A&B	HCF	11 823	1359	3 768 689	5 994 054			
	Kimura 2 (2020)	Japan	2008-2017	>=18	Lab, ICD-10 codes (A04.7)	EIA:GDH, Tox A&B	HCF,ICU	840		239,712				
EU	Novakova1 (2020)	Slovakia	2016 (3mo)	All	Lab	EIA:GDH, Tox A&B,NAAT	unspecified	332	14			1,185,714		RT001,176,017,020,027,049,070
	Novakova2 (2020)	Slovakia	2016 (3mo)	All	Lab	EIA:GDH, Tox A&B,NAAT	HCF	273				1,186,956		RT001,176,017,020,027,049,070

	Novakova3 (2020)	Slovakia	2016 (3mo)	All	Lab	EIA:GDH, Tox A&B,NAAT	CA	45				1,125,000		RT001,176,017,020,027,049,070
EU	Khanafer 1(2016)	France	2007-2014	>=18	Lab+symp	Cul,EIA,GDH, PCR	CA	113			697,468			
	Khanafer 2(2016)	France	2007-2014	>=18	Lab+symp	Cul,EIA,GDH, PCR	ICU	106			697,468			
	Khanafer 3 (2016)	France	2007-2014	>=18	Lab+symp	Cul,EIA,GDH, PCR	IM	54			697,468			
	Khanafer 4 (2016)	France	2007-2014	>=18	Lab+symp	Cul,EIA,GDH, PCR	HCF	448			697,468			
	Khanafer 5(2016)	France	2007-2014	<45 to 65	Lab+symp	Cul,EIA,GDH, PCR	unspecified	286			697,468			
	Khanafer 6 (2016)	France	2007-2014	>65	Lab+symp	Cul,EIA,GDH, PCR	unspecified	304			697,468			
	Khanafer 7 (2016)	France	2007-2014	>=18	Lab+symp	Cul,EIA,GDH, PCR	unspecified	590			697,468			
NA	Kuntz (2016)	US	2005-2012 (6mo)	>=20	ICD code 008.45	No details	HCF	721			54,186			
EU	Alicino (2016)	Italy	2010	No details	Lab+symp	ToxA&B	HO-HCF	19				351,852		
	Alicino (2016)	Italy	2011	No details	Lab+symp	ToxA&B	HO-HCF	32				484,849		
	Alicino (2016)	Italy	2012	No details	Lab+symp	ToxA&B	HO-HCF	74				474,359		
	Alicino (2016)	Italy	2013	No details	Lab+symp	ToxA&B	HO-HCF	125				452,899		
	Alicino (2016)	Italy	2014	No details	Lab+symp	ToxA&B	HO-HCF	131				430,921		
EU	Cioni 1(2016)	Italy	2013-2014 (4mo)	No details	Lab+symp	EIA:GDH, Tox A&B,PCR	HCF IM	103		10780		234,091		ribotype 018,356/607,027,078, 126
	Cioni 2(2016)	Italy	2013-2014 (4mo)	No details	Lab+symp	EIA:GDH, Tox A&B,PCR	HCF NH	20		10780				
WP	Guo (2021)	Hong Kong, China	2015-2019	<44 -64				359		31,797,520				
	Guo (2021)	Hong Kong, China	2015-2019	65->=85				666		31,797,520				
WP	Hung (20210	Taiwan	2011-2013	>=20	Lab+symp	Cul, PCR	IM, HO-HCF	28		461		6611		
EU	Maisa (2019)	Northern Ireland	2012-2016	No details	Lab	GDH,EIA,PCR	CA	1303		9,176,056			64(30d)	RT078, RT026, RT001,RT193.RT023,RT020,RT005,RT015,RT014,RT002

	Maisa (2019)	Northern Ireland	2012-2016	No details	Lab	GDH,EIA,PCR	HCF	1356		9,176,056				RT078, RT026, RT001,RT193.RT023,RT020,RT005,RT015,RT014,RT002
NA	Turner (2023)	US	2017-2022	No details	Lab	NAAT, NAAT/EIA	HO-HCF	1,318				2,657,324		
NA	Ötleş (2023)	US	2020 (4m)	>=18	Lab+symp	EIA, GDH, PCR	HO-HCF	39			2044			
EU	Colomb-Cotinat (2019)	France	2016(3mo)	No detaisls	Lab+symp ESCMID	ToxA&B, PCR	HCF	109				3,056,445		
EM	Meltzer (2019)	Israel	2017(10wks)	No detaisls	Lab	PCR, EIA, CDT	IM, HO-HCF	10			14,286			
EU	Fonesca 1 (2023)	Portugal	2018	>=18	Lab+symp	Tox A&B	HO-HCF	19			14,493		5	
	Fonesca 1 (2023)	Portugal	2018	30-64	Lab+symp	Tox A&B	HO-HCF	2			14,493		5	
	Fonesca 1 (2023)	Portugal	2018	>=65	Lab+symp	Tox A&B	HO-HCF	17			14,493		5	
	Fonesca 2 (2023)	Portugal	2018	>=18	Lab+symp	Tox A&B	CA	11			14,493		2	
	Fonesca 2 (2023)	Portugal	2018	30-64	Lab+symp	Tox A&B	CA	2			14,493		5	
	Fonesca 2 (2023)	Portugal	2018	>=65	Lab+symp	Tox A&B	CA	9			14,493		5	
NA	Hooker (2019)	US	2014-2016	No details	Lab	NAAT(PCR)	HO-HCF	34				42 672		
EM	Kaabia (2024)	Saudi Arabia	2019-2022	All	Lab	PCR	HO-HCF	102				629630		
EU	Nazareth 1 (2022)	Portugal	2017	>=18	Lab+symp	Cul, PCR	HO-HCF	192			190891			
	Nazareth 2 (2022)	Portugal	2017	>=18	Lab+symp	Cul, PCR	CA	174			190891			RT027, RT078/126, RT014, RT106, RT002, RT020, RT017
WP	Kim C1(2024)	Korea	2020-2021	>=20	ICD A047	EIA, Tox A/B, NAAT	HCF	136			17,586	131,824		
	Kim (2024)	Korea	2020-2021	>=20	ICD A047	EIA, Tox A/B, NAAT	HCF	25			13,559	80,113		
	Kim (2024)	Korea	2020-2021	>=20	ICD A047	EIA, Tox A/B, NAAT	HCF	56			17,227	121,422		
	Kim (2024)	Korea	2020-2021	>=20	ICD A047	EIA, Tox A/B, NAAT	HCF	26			10,701	66,131		

	Kim (2024)	Korea	2020-2021	>=20	ICD A047	EIA, Tox A/B, NAAT	HCF	44			12,441	75,565		
	Kim (2024)	Korea	2020-2021	>=20	ICD A047	EIA, Tox A/B, NAAT	HCF	36			10,753	67,555		
	Kim (2024)	Korea	2020-2021	>=20	ICD A047	EIA, Tox A/B, NAAT	HCF	50			11,404	94,363		
	Kim (2024)	Korea	2020-2021	>=20	ICD A047	EIA, Tox A/B, NAAT	HCF	80			14,077	119,642		
	Kim (2024)	Korea	2020-2021	>=20	ICD A047	EIA, Tox A/B, NAAT	HCF	57			26,625	139,856		
	Kim (2024)	Korea	2020-2021	>=20	ICD A047	EIA, Tox A/B, NAAT	HCF	60			10,199	53,920		
	Kim (2024)	Korea	2020-2021	>=20	ICD A047	EIA, Tox A/B, NAAT	HCF	37			9,896	62,122		
	Kim (2024)	Korea	2020-2021	>=20	ICD A047	EIA, Tox A/B, NAAT	HCF	53			4,419	55,152		
	Kim (2024)	Korea	2020-2021	>=20	ICD A047	EIA, Tox A/B, NAAT	HCF	30			7,290	47,851		
	Kim (2024)	Korea	2020-2021	>=20	ICD A047	EIA, Tox A/B, NAAT	HCF	38			7,255	44,294		
	Kim (2024)	Korea	2020-2021	>=20	ICD A047	EIA, Tox A/B, NAAT	HCF	17			6,613	36,979		
	Kim (2024)	Korea	2020-2021	>=20	ICD A047	EIA, Tox A/B, NAAT	HCF	24			1,830	20,866		
	Kim (2024)	Korea	2020-2021	>=20	ICD A047	EIA, Tox A/B, NAAT	HCF	47			6,046	40,252		
	Kim (2024)	Korea	2020-2021	>=20	ICD A047	EIA, Tox A/B, NAAT	HCF	23			5,343	30,664		
	Kim (2024)	Korea	2020-2021	>=20	ICD A047	EIA, Tox A/B, NAAT	CA	15			17,586	131,824		
	Kim (2024)	Korea	2020-2021	>=20	ICD A047	EIA, Tox A/B, NAAT	CA	1			13,559	80,113		
	Kim (2024)	Korea	2020-2021	>=20	ICD A047	EIA, Tox A/B, NAAT	CA	0			17,227	121,422		
	Kim (2024)	Korea	2020-2021	>=20	ICD A047	EIA, Tox A/B, NAAT	CA	2			10,701	66,131		
	Kim (2024)	Korea	2020-2021	>=20	ICD A047	EIA, Tox A/B, NAAT	CA	0			12,441	75,565		
	Kim (2024)	Korea	2020-2021	>=20	ICD A047	EIA, Tox A/B, NAAT	CA	3			10,753	67,555		

	Kim (2024)	Korea	2020-2021	>=20	ICD A047	EIA, Tox A/B, NAAT	CA	4			11,404	94,363		
	Kim (2024)	Korea	2020-2021	>=20	ICD A047	EIA, Tox A/B, NAAT	CA	5			14,077	119,642		
	Kim (2024)	Korea	2020-2021	>=20	ICD A047	EIA, Tox A/B, NAAT	CA	0			26,625	139,856		
	Kim (2024)	Korea	2020-2021	>=20	ICD A047	EIA, Tox A/B, NAAT	CA	5			10,199	53,920		
	Kim (2024)	Korea	2020-2021	>=20	ICD A047	EIA, Tox A/B, NAAT	CA	4			9,896	62,122		
	Kim (2024)	Korea	2020-2021	>=20	ICD A047	EIA, Tox A/B, NAAT	CA	0			4,419	55,152		
	Kim (2024)	Korea	2020-2021	>=20	ICD A047	EIA, Tox A/B, NAAT	CA	7			7,290	47,851		
	Kim (2024)	Korea	2020-2021	>=20	ICD A047	EIA, Tox A/B, NAAT	CA	5			7,255	44,294		
	Kim (2024)	Korea	2020-2021	>=20	ICD A047	EIA, Tox A/B, NAAT	CA	4			6,613	36,979		
	Kim (2024)	Korea	2020-2021	>=20	ICD A047	EIA, Tox A/B, NAAT	CA	5			1,830	20,866		
	Kim (2024)	Korea	2020-2021	>=20	ICD A047	EIA, Tox A/B, NAAT	CA	1			6,046	40,252		
	Kim (2024)	Korea	2020-2021	>=20	ICD A047	EIA, Tox A/B, NAAT	CA	7			5,343	30,664		
EU	Sopena (2022)	Spain	2009-2018	Adults	Lab+symp	EIA, GDH, PCR	unspecified	443				1,419,872	63 (30d)	
NA	Webb (2020)	US	2006-2012	All adults	ICD-9+ Lab	EIA Tox B, NAAT Tox A	ICU	2,356			506,068			
EM	Alzouby (2020)	Saudi Arabia	2015 (6mo)	No details	Lab	ELISA, PCR	unspecified	61	13			137,230		
EU	Van der Werff (2022)	Sweden	2011-2013	No details	ICD-10 code A04.7	No details	HO-HCF	253		719	750			
NA	Turner 1 (2019)	US	2013-2017	No details	Lab	Tox A/B assay, NAAT	HCF	8974			2025 678			NAP1
	Turner 2 (2019)	US	2013-2017	No details	Lab	Tox A/B assay, NAAT	CA	12 280			2025 678			NAP1
NA	Alrawashdeh (2021)	US	2013-2019	No details	Lab	NAAT, Cul, EIA,Cyt	HCF	74 681			24 332 938	109 371 136		

NA	Eberly 1 (2022)	US	2008-2015	>=18	Lab , ICD-9 CM:008.45	EIA,Cul,PCR	HO-HCF	291			474,518			
	Eberly 2(2022)	US	2008-2015	>=65	Lab , ICD-9 CM:008.45	EIA,Cul,PCR	HO-HCF	300			474,518			
LA	Dávila (2017)	Mexico	2011-2015	No details	Lab+symp	PCR, ImmunoCard toxins A&B	HCF	487			288171	1,576,446		R027
NA	Silva 1(2023)	Canada	2015-2020	>=1 to <18	Lab	PCR	HCF	917				1,328,986.00	1(30d)	RT106, RT020, RT014
	Silva 2(2023)	Canada	2015-2020	>=1 to <18	Lab	PCR	CA	279			230,579			
EU	Kachrimanidou 1(2017)	Greece	2014-2015 (9mo)	1-91	Lab	EIA:GDH, Tox A&B,Cul,PCR	unspecified	33		144	13,200		6	RT005,039,126,009,050,137,012,070,202,017,078,220,024,106
	Kachrimanidou 2(2017)	Greece	2014-2015 (9mo)	1-91	Lab	EIA:GDH, Tox A&B,Cul,PCR	CA	9		144				
	Kachrimanidou 3(2017)	Greece	2014-2015 (9mo)	1-91	Lab	EIA:GDH, Tox A&B,Cul,PCR	HCF	24		144				
WP	Mikamo (2020)	Japan	2012-2016	No details	ICD-10 CM: A04.7	No details	unspecified	24,339		4,141,354		100,160,494		
	Mikamo (2020)	Japan	2012-2016	No details	ICD-10 CM: A04.7	No details	HO-HCF	12,624	1436	4,141,354		100,190,476		
NA	Pinzon (2019)	US	2011-2014	No details	ICD-9, Lab	Cul, EIA, PCR	HCF	8423		15 972	2,807,666.00			
EU	Ghosh1 (2024)	UK England	2015-2019	>=18	ICD-10 CM: A04.7	No details	unspecified	36,913	7809	50,722,013				
	Ghosh 1(2024)	UK England	2015-2019	18-64	ICD-10 CM: A04.7	No details	unspecified	9,083	2,003	50,722,013				
	Ghosh1 (2024)	UK England	2015-2019	>=65	ICD-10 CM: A04.7	No details	unspecified	27,830	5,806	50,722,013				
	Ghosh1 (2024)	UK England	2015-2019	>=18	ICD-10 CM: A04.7	No details	HCF	8827	2,665	50,722,013				
	Ghosh1 (2024)	UK England	2015-2019	>=18	ICD-10 CM: A04.7	No details	CA	24,249	3,934	50,722,013				

NA	Miller (2021)	US	2001-2017	>=18	ICD 008.45/A04.7, A04.71, A04.72	No details	HO-HCF	72 038			30 736 764			
NA	Demir (2018)	Canada	2014-2015	No details	Lab	NAAT	HO-HCF	33			2537			
NA	Du (2021)	US	2001-2012	16-90	ICD: 008.45	No details	ICU	1,315			61,532		241	
EU	Teixeira 1 (2021)	Portugal	2014-2017	0-19	ICD-10 CM: A04.7, ICD-9 CM: 008.45	No details	unspecified	49			318,218			
	Teixeira 2 (2021)	Portugal	2014-2017	20-64	ICD-10 CM: A04.7, ICD-9 CM: 008.45	No details	HCF	609			318,218			
	Teixeira 3 (2021)	Portugal	2014-2017	>=65	ICD-10 CM: A04.7, ICD-9 CM: 008.45	No details	HCF	3164			318,218			
	Teixeira 4 (2021)	Portugal	2014-2017	All	ICD-10 CM: A04.7, ICD-9 CM: 008.45	No details	HCF	3822			318,218		636	
EU	Drobnik (2021)	Poland	2016-2018	All	Lab+symp	GDH/EIA, NAAT	HCF	319			183,729		80	
WP	Lee (2021)	Taiwan	2017-2018	>=20	Lab+symp	GDH, PCR	ICU	23				21495		
WP	Johnston 1 (2022)	New Zealand	2018-2019	>2	Lab+symp	GDH, EIA Tox A&B	HCF	23		855		62162		
	Johnston 2 (2022)	New Zealand	2018-2019	>2	Lab+symp	GDH, EIA Tox A&B	CA	9		855				
EU	Demir (2021)	Turkey	2015-2016	2 to 18	Lab + symp	EIA	HO-HCF	19		1,971	3,172	21,520		
EU	Khanafer (2018)	France	2011-2014	>=18	Lab+symp	EIA,GDH, PCR	HCF	233		945		749,516		
WP	Yang (2020)	China	2014-2019	No details	Lab+symp	PCR	HCF	336				475,920		
EU	Roncarati(2017)	Italy	2015	No details	Lab	ToxA&B	IM	503				882,456		
	Roncarati(2017)	Italy	2015	No details	Lab	ToxA&B	ICU	17				1,100,000		
	Roncarati(2017)	Italy	2015	No details	Lab	ToxA&B	LCF	327				73,913		
	Roncarati H1(2017)	Italy	2015	No details	Lab	ToxA&B	unspecified	264				1,100,000		
	Roncarati H2(2017)	Italy	2015	No details	Lab	ToxA&B	unspecified	125				462,963		

	Roncarati H3(2017)	Italy	2015	No details	Lab	ToxA&B	unspecified	127				276,087		
	Roncarati H4(2017)	Italy	2015	No details	Lab	ToxA&B	unspecified	36				200,000		
	Roncarati H5(2017)	Italy	2015	No details	Lab	ToxA&B	unspecified	130				151,163		
	Roncarati H6(2017)	Italy	2015	No details	Lab	ToxA&B	unspecified	53				112,766		
	Roncarati H7(2017)	Italy	2015	No details	Lab	ToxA&B	unspecified	67				109,836		
	Roncarati H8(2017)	Italy	2015	No details	Lab	ToxA&B	unspecified	28				68,292		
	Roncarati H9(2017)	Italy	2015	No details	Lab	ToxA&B	unspecified	112				62,921		
NA	Hunter C1 (2016)	US	2012	No details	Lab	NAAT	NH	478		853,571				RT027,RT002,RT106,RT078
	Hunter C2 (2016)	US	2012	No details	Lab	NAAT	NH	758		2,501,650				
	Hunter C3 (2016)	US	2012	No details	Lab	NAAT	NH	219		820,225				
	Hunter C4 (2016)	US	2012	No details	Lab	NAAT	NH	637		3,769,230				
	Hunter C5(2016)	US	2012	No details	Lab	NAAT	NH	475		830,420				
	Hunter C6 (2016)	US	2012	No details	Lab	NAAT	NH	21		244,186				
	Hunter C7(2016)	US	2012	No details	Lab	NAAT	NH	406		664,484				
	Hunter C8(2016)	US	2012	No details	Lab	NAAT	NH	345		738,758				
	Hunter C9 (2016)	US	2012	No details	Lab	NAAT	NH	13		224,138				
	Hunter C10(2016)	US	2012	No details	Lab	NAAT	NH	151		639,831				
EM	Atamna (2016)	Israel	2009-2014	>=18	Lab+symp	GDH, PCR	ICU	499			55,506			
EU	Sandell (2016)	Sweden	2009-2011	>18	Lab+symp	Cul, Cyt, PCR	HCF	157		2150				
WP	Lee (2016)	Taiwan	2013-2014	>=20	Lab+symp	Cul, PCR, EIA Tox A&B	ICU	14			1551			

Notes: LA: Latin America, NA: North America, EU: Europe, WP: Western Pacific, EM: Eastern Mediterranean (i) EIA: Enzyme Immunoassay, Tox: Toxin, &: and, /: orCyt: Cytotoxin, GDH: glutamate dehydrogenase, Cul: Culture, PCR: polymerase chain reaction, NAAT: Nucleic Acid Amplification Test, ELISA: Enzyme-Linked Immunosorbent Assay. Lab: Laboratory confirmation, Symp: symptoms confirmation, including at least one of the following diarrhoeal disease, endoscopic or histologic features.

Supplementary Table 2. Assessment of Study Quality for *Clostridium difficile* Infection

Study	Title/Abstract	Background	Objectives	Study Design	Setting	Participants	Data Sources	Variables	Bias	Study Size	Statiscal methods	Participants	Descriptive data	Outcome Data	Main results	Interpretation	Limitations	SCORE	GRADING
Govoni (2021)	Yes	Yes	Maybe	Yes	Yes	Yes	Yes	Yes	No	No	Maybe	Yes	Yes	Yes	Maybe	Yes	Yes	13.5	Moderate Quality
Lauda-Maillen (2019)	Yes	Yes	Yes	Yes	Yes	Yes	Yes	Yes	No	No	Maybe	Yes	Maybe	Yes	Maybe	Yes	Yes	13.5	Moderate Quality
Olsen (2019)	Yes	Yes	Yes	Yes	Yes	Yes	Yes	Yes	No	No	Yes	Yes	Maybe	Yes	Maybe	Yes	Yes	14	High Quality
Miller (2016)	Yes	Yes	Yes	Yes	Yes	Yes	Yes	Yes	No	No	Yes	Yes	Yes	Yes	Maybe	Yes	Yes	14.5	High Quality
Longtin (2016)	Yes	Yes	Yes	Yes	Yes	Yes	Yes	Yes	No	No	Yes	Yes	Maybe	Yes	Yes	Yes	Yes	14.5	High Quality
Miller (2016)	Yes	Yes	Yes	Yes	Yes	Yes	Yes	Yes	No	No	Yes	Maybe	Maybe	Yes	Yes	Yes	Yes	14	High Quality
Alkhawaja (2024)	Yes	Yes	Yes	Yes	Yes	Yes	Yes	Yes	No	No	Yes	Maybe	Maybe	Yes	Yes	Yes	Yes	14	High Quality
Karaaslan (2016)	Yes	Yes	Yes	Yes	Yes	Yes	Yes	Yes	No	No	Yes	Yes	Maybe	Yes	Yes	Yes	Yes	14.5	High Quality
Barbosa-Martins S (2022)	Yes	Yes	Yes	Yes	Yes	Yes	Yes	Yes	No	No	Yes	Yes	Maybe	Yes	Yes	Yes	Yes	14.5	High Quality
Kotila (2016)	Maybe	Yes	Yes	Maybe	Yes	Yes	Yes	Yes	Maybe	Yes	Yes	Yes	Yes	Yes	Yes	Yes	Yes	15.5	High Quality
Katz S (2018)	Yes	Yes	Yes	Yes	Yes	Yes	Yes	Yes	Maybe	Yes	Yes	Yes	Yes	Yes	Yes	Yes	Yes	16.5	High Quality
Chen (2017)	Maybe	Yes	Yes	Yes	Yes	Yes	Yes	Yes	Yes	Yes	Yes	Yes	Yes	Yes	Yes	Yes	Yes	16.5	High Quality
Xu (2017)	Maybe	Yes	Yes	Yes	Yes	Yes	Yes	Yes	No	No	Yes	Yes	Yes	Yes	Yes	Yes	Yes	14.5	High Quality
Kimura (2020)	Yes	Yes	Yes	Yes	Yes	Yes	Yes	Yes	Yes	Yes	Yes	Yes	Yes	Yes	Yes	Yes	Yes	17	High Quality
Novakova S (2020)	Maybe	Yes	Yes	Maybe	Yes	Maybe	Yes	Maybe	No	No	Yes	Maybe	Yes	Yes	Yes	Yes	Yes	12.5	Moderate Quality
Khanafer S (2016)	Yes	Yes	Yes	Yes	Yes	Yes	Yes	Yes	No	No	Yes	Yes	Yes	Yes	Yes	Yes	Yes	15	High Quality
Kuntz (2016)	Yes	Yes	Yes	Yes	Maybe	Yes	Yes	Maybe	No	No	Yes	Yes	Yes	Yes	Yes	Yes	Yes	14	High Quality
Alicino (2016)	Maybe	Yes	Yes	Yes	Yes	Yes	Yes	Yes	No	No	Yes	Yes	Yes	Yes	Yes	Yes	Yes	14.5	High Quality
Cioni (2016)	Yes	Yes	Yes	Yes	Yes	Yes	Yes	Yes	No	No	Yes	Yes	Yes	Yes	Yes	Yes	Yes	15	High Quality
Guo (2021)	Maybe	Yes	Yes	Maybe	Yes	Yes	Yes	Yes	No	No	Yes	Yes	Yes	Yes	Yes	Yes	Yes	14	High Quality
Hung (2021)	Yes	Yes	Yes	Yes	Yes	Yes	Yes	Yes	Maybe	No	Yes	Yes	Yes	Yes	Yes	Yes	Yes	15.5	High Quality
Maisa (2019) S	Maybe	Yes	Yes	Maybe	Yes	Yes	Yes	Yes	No	Yes	Yes	Yes	Yes	Yes	Maybe	Yes	Yes	14.5	High Quality
Turner (2023)	Maybe	Yes	Yes	Yes	Maybe	Maybe	Yes	Yes	No	No	Yes	Yes	No	Yes	Maybe	Yes	Yes	12	Moderate Quality
Ötleş (2023) S	Yes	Yes	Yes	Maybe	Maybe	Yes	Yes	Yes	No	Yes	Yes	Yes	Yes	Yes	Maybe	Yes	Yes	14.5	High Quality
Colomb-Cotinat (2019) S	Yes	Yes	Yes	Maybe	Yes	Yes	Yes	Maybe	No	No	Yes	Yes	No	Yes	Maybe	Yes	Yes	12.5	Moderate Quality
Meltzer (2019) S	Maybe	Yes	Yes	Maybe	Maybe	Yes	Yes	Yes	No	No	Yes	Yes	Yes	Yes	Maybe	Yes	Yes	13	Moderate Quality

Fonseca (2023)	Maybe	Yes	Yes	Yes	Maybe	Yes	Yes	Yes	No	No	Yes	Yes	Yes	Yes	Maybe	Yes	Yes	13.5	Moderate Quality
Hooker (2019)	Yes	Yes	Yes	Yes	Yes	Maybe	Maybe	Maybe	No	No	Yes	Yes	Maybe	Yes	Maybe	Yes	Yes	12.5	Moderate Quality
Kaabia (2024) S	Yes	Yes	Yes	Yes	Yes	Yes	Yes	Yes	No	No	No	Yes	Yes	Yes	No	Yes	Yes	13	Moderate Quality
Nazareth (2022) S	Yes	Yes	Yes	Yes	Maybe	Yes	Yes	Yes	No	No	Yes	Yes	Yes	Yes	Yes	Yes	Yes	14.5	High Quality
Kim (2024) S	Yes	Yes	Yes	Yes	Yes	Yes	Yes	Maybe	No	No	Yes	Yes	Maybe	Yes	Maybe	Yes	Yes	13.5	Moderate Quality
Sopena (2022)	Yes	Yes	Yes	Yes	Yes	Yes	Yes	Yes	No	No	Yes	Yes	Yes	Yes	Yes	Yes	Yes	15	High Quality
Webb (2020)	Yes	Yes	Yes	Yes	Yes	Yes	Yes	Yes	Maybe	No	Yes	Yes	Yes	Yes	Yes	Yes	Yes	15.5	High Quality
Alzouby (2020) S	Yes	Yes	Yes	Yes	Yes	Yes	Maybe	Maybe	No	No	Yes	Yes	Maybe	Yes	No	Yes	Yes	12.5	Moderate Quality
Van der Werff (2022)	No	Yes	Yes	Yes	Yes	Yes	Yes	Maybe	No	Yes	Yes	Yes	Yes	Yes	Yes	Yes	Yes	14.5	High Quality
Turner (2019)	Yes	Yes	Yes	Yes	Maybe	No	Yes	Maybe	No	No	Yes	Yes	Yes	Yes	Yes	Yes	Yes	13	Moderate Quality
Alrawashdeh (2021)	Yes	Yes	Yes	Yes	Yes	Yes	Yes	Yes	No	No	Yes	Yes	Yes	Yes	Yes	Yes	Yes	15	High Quality
Eberly (2022)	Yes	Yes	Yes	Yes	Maybe	Yes	Yes	Yes	No	No	Maybe	Yes	Yes	Yes	Yes	Yes	Yes	14	High Quality
Dávila (2017)	Yes	Yes	Yes	Yes	Yes	Yes	Yes	Yes	No	No	Yes	Yes	Yes	Yes	Maybe	Yes	Yes	14.5	High Quality
Silva S(2023)	No	Yes	Yes	Yes	Maybe	Yes	Yes	Maybe	No	No	Yes	Yes	Yes	Yes	Maybe	Yes	Yes	12.5	Moderate Quality
Kachrimanidou (2017)	Yes	Yes	Yes	Yes	Yes	Yes	Yes	Maybe	No	Yes	Yes	Yes	Yes	Yes	No	Yes	Yes	14.5	High Quality
Mikamo (2020)	Yes	Yes	Yes	Yes	Yes	Yes	Yes	Yes	No	No	Yes	Yes	Yes	Yes	Yes	Yes	Yes	15	High Quality
Pinzon (2019)	Yes	Yes	Yes	Yes	Yes	Yes	Yes	Yes	No	No	Yes	Yes	Yes	Yes	Yes	Yes	Yes	15	High Quality
Ghosh (2024)	Yes	Yes	Yes	Yes	Yes	Yes	Yes	Yes	No	No	Yes	Yes	Yes	Yes	Yes	Yes	Yes	15	High Quality
Miller (2021)	Maybe	Yes	Yes	Yes	Maybe	Yes	Yes	Yes	No	Yes	Yes	Yes	Yes	Yes	Maybe	Yes	Yes	14.5	High Quality
Demir (2018)	Yes	Yes	Yes	Yes	Yes	Yes	Yes	Yes	No	No	Yes	Yes	Yes	Yes	Yes	Yes	Yes	15	High Quality
Du (2021)	Maybe	Yes	Yes	Yes	Yes	Yes	Yes	Yes	No	No	Yes	Yes	Yes	Yes	Maybe	Yes	Yes	14	High Quality
Teixeira (2021)	Maybe	Yes	Yes	Yes	Yes	Yes	Yes	Yes	No	No	Yes	Yes	Yes	Yes	Maybe	Yes	Yes	14	High Quality
Drobnik (2024)	Maybe	Yes	Yes	Maybe	Yes	Yes	Yes	Yes	No	No	Yes	Yes	Yes	Yes	Maybe	Yes	Yes	13.5	Moderate Quality
Lee (2021)	Maybe	Yes	Yes	Maybe	Yes	Yes	Yes	Yes	No	No	Yes	Yes	Yes	Yes	Maybe	Yes	Yes	13.5	Moderate Quality
Johnston (2022)	Yes	Yes	Yes	Yes	Maybe	Yes	Yes	Yes	No	No	Yes	Yes	Yes	Yes	Maybe	Yes	Yes	14	High Quality
Demir (2021)	Yes	Yes	Yes	Yes	Yes	Yes	Yes	Yes	No	No	Yes	Yes	Yes	Yes	Maybe	Yes	Yes	14.5	High Quality
Khanafer (2018)	Yes	Yes	Yes	Yes	Yes	Yes	Yes	Maybe	No	No	Yes	Yes	Yes	Yes	No	Yes	Yes	13.5	Moderate Quality
Yang (2020)	Maybe	Yes	Yes	Yes	Yes	Yes	Yes	Yes	No	Yes	Yes	Yes	Maybe	Yes	Maybe	Yes	Yes	14.5	High Quality
Roncarati (2017)S	Yes	Yes	Yes	Yes	Yes	Yes	Yes	Yes	Maybe	Yes	Yes	Yes	Maybe	Yes	Maybe	Yes	Yes	15.5	High Quality
Hunter (2016) S	Maybe	Yes	Yes	Maybe	Yes	Yes	Yes	Yes	No	Yes	Yes	Yes	Yes	Yes	Yes	Yes	Yes	15	High Quality

Atamna (2016)	Yes	Yes	Yes	Yes	Yes	Yes	Yes	Yes	No	No	Yes	Yes	Yes	Yes	Yes	Yes	Yes	15	High Quality
Sandell (2016)	Maybe	Yes	Yes	Mayb e	Yes	Yes	Yes	Yes	No	No	Yes	Yes	Yes	Yes	No	Yes	Yes	13	Moderate Quality
Lee (2016)	Maybe	Yes	Yes	Yes	Yes	Yes	Yes	Yes	No	No	Yes	Yes	Yes	Yes	No	Yes	Yes	13.5	Moderate Quality
